# Cutaneous Papillomaviruses and Non-melanoma Skin Cancer: Causal Agents or Innocent Bystanders?

**DOI:** 10.3389/fmicb.2018.00874

**Published:** 2018-05-02

**Authors:** Daniel Hasche, Sabrina E. Vinzón, Frank Rösl

**Affiliations:** ^1^Division of Viral Transformation Mechanisms, Research Program “Infection, Inflammation and Cancer”, German Cancer Research Center, Heidelberg, Germany; ^2^Laboratory of Molecular and Cellular Therapy, Fundación Instituto Leloir, IIBBA-CONICET, Buenos Aires, Argentina

**Keywords:** cutaneous papillomaviruses, hit-and-run mechanism, *Mastomys coucha*, causality, skin cancer, animal models, NMSC

## Abstract

There is still controversy in the scientific field about whether certain types of cutaneous human papillomaviruses (HPVs) are causally involved in the development of non-melanoma skin cancer (NMSC). Deciphering the etiological role of cutaneous HPVs requires – besides tissue culture systems – appropriate preclinical models to match the obtained results with clinical data from affected patients. Clear scientific evidence about the etiology and underlying mechanisms involved in NMSC development is fundamental to provide reasonable arguments for public health institutions to classify at least certain cutaneous HPVs as group 1 carcinogens. This in turn would have implications on fundraising institutions and health care decision makers to force – similarly as for anogenital cancer – the implementation of a broad vaccination program against “high-risk” cutaneous HPVs to prevent NMSC as the most frequent cancer worldwide. Precise knowledge of the multi-step progression from normal cells to cancer is a prerequisite to understand the functional and clinical impact of cofactors that affect the individual outcome and the personalized treatment of a disease. This overview summarizes not only recent arguments that favor the acceptance of a viral etiology in NMSC development but also reflects aspects of causality in medicine, the use of empirically meaningful model systems and strategies for prevention.

## The Problem – a Serious and Common Disease: Non-Melanoma Skin Cancer

Non-melanoma skin cancer is the most frequent cancer in fair-skinned individuals worldwide and its incidence has increased during the last three decades (Welsh et al., 2011; Lomas et al., 2012). There are up to 3 million new cases every year, whereby women below 45 years are even more affected (Al-Dujaili et al., 2017). Based on different UV exposure rates, there are also geographical variations, with the highest incidence of NMSC in Australia (Lomas et al., 2012). Although the metastatic potential and the mortality rates in immunocompetent individuals are low in comparison to malignant melanomas, NMSC has a strong impact on the quality of life of the affected persons and the financial burden on health care systems. Taking the United States as an example, total annual expenses for NMSC medical care amounts to 650 million dollars (Apalla et al., 2017). Hence, there should be a quite obvious public health interest to understand and to prevent this cancer entity.

Non-melanoma skin cancer principally refers to cancer derived from keratinocytes (Small et al., 2016) and can be further divided into basal cell carcinomas (BCCs) and squamous cell carcinomas (SCCs), with relative frequencies of 80 and 20%, respectively (Eisemann et al., 2014). While BCCs are not very different in their biological behaviors, SCCs show broad histopathological diversities that are associated with markedly different clinical outcomes. Depending on the immune status of the patient, these may range from indolent tumors with low metastatic capacity to aggressive tumors with high invasive potential. Approximately 97% of invasive SCCs are found in association with malignant progression of an actinic keratosis (AK), considered as a precursor of SCCs. These lesions can display erythematous, scaling, and rough patches. They can either spontaneously regress, persist as a benign AK or progress to an invasive SCC where cells infiltrate the basement membrane into the dermis (reviewed in Yanofsky et al., 2011). TP53 is found to be mutated in dysplastic AKs, indicating that functional loss is a very early event, connected with loss of chromosomes including the cell cycle regulator p16INK4A (Ratushny et al., 2012). To define some general histopathological features, a committee of European dermatologists has recently identified telangiectasia, atrophy, and pigmentation disorders as the most reliable markers for AKs (Dreno et al., 2017).

Other clinically common lesions are keratoacanthomas (KAs) which can form spontaneously but, after a proliferative and subsequent resting phase, have the tendency to regress, apparently due to apoptosis. Histologically, a KA is an exo-/endophytic growing squamous proliferative tumor with a characteristic central keratin plug, surrounded by epidermal lips, forming a relatively well-defined symmetrical structure (Takai, 2017). Of note, approximately 25% of KAs can develop into SCCs (Sanchez Yus et al., 2000). Although still controversially discussed in terms of whether a KA is a variant of SCC, other dermatologists believe it represents a self-resolving benign epidermal lesion (reviewed in Savage and Maize, 2014). This illustrates the current discrepancies between histopathological phenotypes and deep sequencing DNA profiles of respective specimens, since KAs and SCCs are distinct entities at least by their unique molecular gene signatures (Ra et al., 2015). Nonetheless, according to the “World Health Organization Classification of Tumors” as a guideline for pathologists in diagnostics, KAs are still regarded as a well-differentiated SCC (LeBoit et al., 2006).

Another thought-provoking inquiry that has clinical significance in the context of NMSC pathogenesis emerged during the last years, when patients with advanced metastatic melanomas were treated with BRAF inhibitors (BRAFi) (e.g., vemurafenib or dabrafenib) (reviewed in Gibney et al., 2013). The off-target outcome of such drugs results in deleterious dermatological side effects, histopathologically ranging from *verrucal keratosis*, hair follicle changes, plantar hyperkeratosis, KAs and finally cutaneous SCCs (Anforth et al., 2013). Vemurafenib also has a photosensitizing activity (Boussemart et al., 2013), consistent with the finding that most SCCs can be detected at chronically sun-damaged skin regions (Hassel et al., 2015).

## Endogenous and Environmental Risk Factors for NMSC

### The Individual Genotype

Cancer in general is a multi-factorial disease and as individual as the patient, a reasonable concept that is culminating in the contemporary discourse of personalized medicine (reviewed in Mavropoulos et al., 2014; Harwood et al., 2016). Referring to this notion, meta-analyses of the current literature have deciphered several risk factors that are either distinct in terms of an inherent genetic predisposition of an individual patient and/or caused by environmental factors (reviewed in Wang et al., 2014; Belbasis et al., 2016). Individual risk factors for NMSC are age, family history, pigmentation, polymorphisms within genes encoding IL10, IL4R, TNF, or TNFR2 (Welsh et al., 2011), several inborn genetic disorders like *Xeroderma pigmentosum* (Black, 2016), the basal cell nevus syndrome (reviewed in Bresler et al., 2016) or the WHIM (Wart, Hypogammaglobulinemia, Infection, and Myelokathexis) syndrome, an autosomal dominant inheritance immune deficiency, harboring mutations in the chemokine receptor CXCR4 (Chow et al., 2010; Meuris et al., 2016). Such patients show a high susceptibility for both cutaneous and mucosal HPVs. Multiple warts can occur on hands and feet, but also genital condylomata acuminata can be found (Leiding and Holland, 2012). In the case of patients suffering from the rare hereditary disease *Epidermodysplasia verruciformis* (EV), mutations within at least two genes of homologous transmembrane channel-like (TMC) proteins, TMC6 (EVER1) and TMC8 (EVER2) were described (Lazarczyk et al., 2009, 2012). These proteins are involved in zinc homeostasis and are not only expressed in keratinocytes, but also in lymphocytes, suggesting additional functions in the immune response (reviewed in Kalinska-Bienias et al., 2016). Due to their interaction with the Zn^2+^ transporter protein ZnT-1, TMC6/8 can further modulate MAP kinases and in turn AP-1, a transcription factor involved in many signal transduction pathways (reviewed in Lazarczyk and Favre, 2008).

### Immune Status

Another important risk factor is iatrogenic immunosuppression as SCCs appear 65–250 times more frequently in OTRs compared to the general population (reviewed in Nindl and Rösl, 2008; Vinzón and Rösl, 2015). Moreover, the frequency of tumor formation correlates with the extent and duration of immunosuppression (reviewed in Euvrard et al., 2003; Hampton, 2005). It is estimated that up to 40% of OTRs will develop skin cancers within the first 10 years of transplantation and up to 80% after 20 years (Rangwala and Tsai, 2011). Although early detection of NMSC evidently improves the overall survival rate, a 20 year follow-up study including more than 85,000 patients showed that OTRs have a much worse prognosis and a higher mortality rate as compared to immunocompetent individuals (Acuna et al., 2016). The exact physiological reason is still unexplained, but it is assumed that tumors are simply more aggressive in an immunosuppressive environment since substances like cyclosporine both promote tumor invasion and favor neovascularization (reviewed in Geissler, 2015). Immunosuppressive drugs apparently also impair the DDR and counteract p53-dependent cellular senescence (Wu et al., 2010; Kuschal et al., 2012). Since SCCs develop mostly within sun-exposed areas, UV exposure is immune compromising (reviewed in Nakad and Schumacher, 2016). Despite the obvious increase of NMSC, population-based studies of skin cancer mortality after organ transplantation and in immunocompetent individuals are still scant (John et al., 2016). This is mainly due to geographic variability of incidence rates and, most importantly, the exclusion of NMSC from central cancer registries records (reviewed in Apalla et al., 2017) as recently noticed in the “*European Code against Cancer: Infection and Cancer*” (Villain et al., 2015). Such an obvious gap in cancer documentation should be filled as exemplified by initiatives such as the “Keratinocyte Carcinoma Consortium” (Madeleine et al., 2017), because NMSC incidence is increasing worldwide and caused by daily and life-long risk factors such as UV exposure and skin infections (Garrett et al., 2016; Prasad and Katiyar, 2017).

### Sun Exposure

Another major environmental risk factor that has to be taken into account for the development of NMSC is the cumulative lifelong exposure to UV light, as well as sunburns in youth (Kennedy et al., 2003). UV light is divided into three categories according to its energy (wave length), which are UVA (320–400 nm), UVB (290–320 nm), and UVC (100–290 nm). UVC is almost completely filtered by the atmospheric ozone layer, which also shields up to 90% of UVB radiation. Thus, only UVA and UVB play roles as risk factors. Although not penetrating in the skin as deep as UVA, due to its higher energy output, UVB is more harmful and can damage cells heavily (reviewed in Seebode et al., 2016). UVB has also additional deleterious side-effects on the immune system, for example, by perturbing the function of antigen-presenting cells and in turn increasing a local immunodeficiency (reviewed in Prasad and Katiyar, 2017). Thus, cumulative UV exposure can contribute to skin tumor development by impairing immune surveillance of the skin (Maglennon et al., 2014). UV has also direct effects on the DNA, leading to a covalent joining of adjacent pyrimidines to form cyclobutane pyrimidine dimers (CPDs). Due to the misincorporation of an adenine at the position complementary to the UV DNA damage, CPDs predominantly lead to C→T transitions (≥60%) and rarely to CC→TT transitions (5%), leaving characteristic signatures within the DNA that can be detected in the landscape of SCCs upon whole genome sequencing (reviewed in Brash, 2015).

However, it is still not clear how to functionally rank the significance and frequencies of such mutations since the appearance of NMSC is, like other forms of cancer, the result of a multi-step process in which the temporal/spatial order and the impact on tumor formation cannot retrospectively be defined. Moreover, comparatively little is known about the temporal combinatory linkage of altered gene expression within a cellular network, thus contributing to the individual manifestation of a tumor (Krogan et al., 2015). Notably, deep-sequencing analyses of normal skin sections of persons with different histories of sun exposures even revealed identical UV specific signatures in their genome with exactly the same genes affected as later found in SCCs or BCCs (Martincorena et al., 2015). The reasons why such mutations are tolerated in normal cells suggest a counter-selective environment consisting of stromal, endothelial, and inflammatory components that prevent the outgrowth of clones finally forming a tumor. Considering recent concepts, cancer is not simply a linear route, but a highly flexible and emergent adaptation process of certain cells where clonal expansion is following according to evolutionary principles that limit the predictability of therapeutic outcomes (reviewed in Greaves, 2015; Lipinski et al., 2016; Davis et al., 2017).

### Cutaneous Papillomaviruses

Since individuals are “open systems,” the most critical exogenous risk factor for NMSC is not only UV exposure, but also infections with papillomaviruses. These target keratinocytes of the skin and mucosa of different vertebrate species, including humans. Up to date more than 200 human papillomavirus (HPV) types are known according to the PaVE database^1^ (Van Doorslaer et al., 2017), which are associated with different clinical manifestations. Genital HPVs cause diverse lesions ranging from benign warts (for “low-risk” HPV types) to different malignancies (for “high-risk” types) of which cervical cancer is the most prominent (zur Hausen, 2002; McLaughlin-Drubin et al., 2012).

On the other hand, there is also a wide range of cutaneous HPV types that have been associated with diverse skin diseases (reviewed in Grce and Mravak-Stipetic, 2014). Including more than 100 healthy human volunteers in a broad study of meta-genomic analyses, whole-genome shotgun sequencing revealed an overall HPV prevalence of 69% in the skin, followed by the vagina (42%), the mouth (30%), and the gut (17%) (Ma et al., 2014). Another study even demonstrated that more than 95% of all viral sequences detected in skin samples belong to the papillomavirus family, mostly to the beta-and gamma genera. Here, also co-infections could be discerned, which leads to speculation about potential temporal inclusion/exclusion interaction mechanisms between different HPV types in the same cell that may modulate the immune response and in turn viral persistence (Bzhalava et al., 2014).

High prevalence of cutaneous HPV can be already detected in skin of infants and young children, indicating that viral exposure occurs very early in life (Antonsson et al., 2003). Recurrent types are HPV2, 7, 27, 57 (alpha genus), HPV23 and 75 (beta genus), HPV4 and 65 (gamma genus) and HPV1 (mu genus) that typically cause only benign lesions such as common warts (*verrucae vulgaris*), plantar warts (*verrucae plantaris*), and flat warts (*verruca plana*) (Jablonska et al., 1997; Cardoso and Calonje, 2011; Bruggink et al., 2012) which usually spontaneously regress (van Haalen et al., 2009). However, they can represent a serious problem for immunocompromised individuals, especially OTRs, who frequently suffer from confluent wart formation all over the body. The prevalence within this population ranged from 48 to 92% in the first 5 years after immunosuppression (Jablonska et al., 1997; Wang et al., 2014). The HIM study (“cutaneous HPV infection in healthy men”) reported a different median time of incidence (=newly diagnosed in skin) and duration of persistence of beta and gamma HPV infections ranging between 6–8 and 6–11 months, respectively. Older volunteers showed a significant higher association with prevalent and persistent HPV infections (Hampras et al., 2014). However, merely monitoring the skin for the presence of a viral DNA does not allow any conclusions about an ongoing infection and the exposure of viral antigens to the immune system. Such questions can only be answered when combined with immunological studies to get estimations about the rates of seroconversion and the serodynamics of cutaneous HPV infections (Rahman et al., 2016), an important criteria when preclinical models are used (see below).

### “High Risk” Cutaneous HPVs?

The term “infection” indicates the presence of a microbe which can be pathogenic, commensal or opportunistic (reviewed in Casadevall and Pirofski, 2000). Hence, considering the individual complexity of the human skin virome that in turn also represents an interactome with a defined microbial environment (Hannigan et al., 2015), it is a huge challenge of modern biomedicine to dissect such networks in terms of functionality and causality. Indeed, the skin is an open ecosystem, colonized by a broad spectrum of bacteria and viruses whose landscape and impact on local immunity change during their individual life span and by environmental factors (Belkaid and Segre, 2014). We still do not understand how these different constituents dynamically interact with other microorganisms colonizing the skin, what the selective advantage of such a commensalism is, how the virome in turn affects the transcriptome of the skin (Edqvist et al., 2015), form the individual immunophenotype and how these properties vary with respect of individual skin types, under immunosuppression or UV exposure (Virgin, 2014). The categories commensal, pathogenic or opportunistic are helpful notions in the context of tumor viruses (Moore and Chang, 2017). Accordingly, commensal tumor viruses (e.g., EBV or cutaneous HPVs), are almost ubiquitously spread and usually do not cause symptoms. Contrarily, pathogenic tumor viruses are not so frequent and acquired, for instance via sexual intercourse or blood transfusion (e.g., high-risk mucosal types of HPV or Hepatitis B virus, HBV). Due to their ubiquitous prevalence in the general healthy population, there is still skepticism whether cutaneous HPVs are causally involved in NMSC development. However, there are many epidemiological and mechanistic *in vitro/in vivo* data showing increasing evidence that at least some commensal HPVs represent an essential, if not even a decisive factor for this type of skin cancer, especially for SCC (Madeleine et al., 2013; Wang et al., 2014; Chahoud et al., 2016).

Historically, the oncogenic potential of beta-type HPV infection (e.g., HPV5 and HPV8) in NMSC has been originally identified in patients suffering from EV, characterized by an increased susceptibility to viral infection (Orth, 2006). The oncogenic and transforming capacity of the EV-HPVs has not only been shown in transgenic mouse models (Schaper et al., 2005; Pfefferle et al., 2008; Viarisio et al., 2011), but also in organotypic raft cultures under *in vitro* conditions (Borgogna et al., 2012; Heuser et al., 2016b; Marthaler et al., 2017). Beta-type papillomaviruses were also detected in NMSCs of non-EV patients, although the viral loads are usually very low. It is assumed that the reservoirs for latent infection are hair follicle stem cells (Hufbauer et al., 2013). Different studies have reported HPV DNA in 30–50% of NMSCs from immunocompetent persons (Harwood et al., 2006), whereas in immunosuppressed patients this figure goes up to 90% (Harwood et al., 2000; Arnold and Hofbauer, 2012). Transcriptionally active beta-type HPVs can be found in premalignant lesions such as AKs (Borgogna et al., 2014) but also in normal skin and plucked eyebrow hairs suggesting a functional correlation between the presence of viral DNA and an increased risk of NMSC (Neale et al., 2013). UV exposure *per se* can also directly stimulate viral transcription of the EV-types HPV5 and HPV8 (Akgül et al., 2005b), while other cutaneous types such as HPV38 E6/E7, for instance, can block UV-induced expression of the toll-like receptor 9 (TLR9), normally responsible for endosomal sensoring of exogenic DNA (Pacini et al., 2017). This shows the complexity of a virus–host interaction, demonstrating several modes of action by different HPV types to interfere with signal transduction pathways of the host cell. Moreover, in contrast to anogenital tumors, there are also SCCs that either completely lack HPV or not all cells are virus-positive (Neale et al., 2013), indicating that viral oncoprotein expression is not necessary to maintain a malignant phenotype (reviewed in Arron et al., 2011; Hufbauer and Akgül, 2017).

## Some Reflections About the “Causality” of Infections

The lack of HPV in cancerous skin lesions is in apparent contradiction to the so-called Henle-Koch’s postulates that were traditionally used as criteria for “causality” of an infection (reviewed in Inglis, 2007). However, one should keep in mind that the conception of causality is a term historically referring to physical properties of the inanimate matter and not to living systems. Physicists deal with reproducible regular events and experimental settings, while cancer research is investigating “anomalous” scenarios, sometimes rare disorders in terms of cancer development which are, as aforementioned, multi-factorial and individually shaped. Hence, the simple detection of an infection or frequent mutations found in genetically non-predisposed persons cannot be considered as “causal,” because they are only contingent and the individual clinical outcome is determined by many additional factors (reviewed in Greaves, 2015). More correct in an epistemic sense is the word “etiology,” since this term has a more suitable connotation, reflecting more the complexity of an organism and not implying necessarily a direct and quite obvious causal relationship.

Nonetheless, in principle the Henle-Koch’s postulates state that an infectious agent should be consistently detected in the respective lesions, it should be isolated and its re-inoculation should induce the same symptoms or clinical manifestations. However, these postulates have their limitations, because they are not considering multi-factorial events in the development of a disease (reviewed in Edwards and Rohwer, 2005; Virgin, 2014; O’Malley, 2016). Moreover, they also do not reflect asymptomatic, latent or persistent infections, all states in which HPV DNA can exist (Sudenga and Shrestha, 2013). Anecdotally, Robert J. Huebner, Chief of the Laboratory of RNA Tumor Viruses (NCI) recognized this inadequacy by referring it as the “The Virologist’s Dilemma” (Huebner, 1957). As a consequence, he paved the way for the inclusion and particularly the acceptance of epidemiological data to corroborate the involvement of a virus as a cause for a specific disease (Vonka, 2000).

The progress in molecular technologies and immunological methods (Falkow, 2004) therefore led to a revision of these criteria, by both including epidemiological and molecular data as well as terminologies like “plausibility” (=whether a causal relationship makes biological sense) and “coherence” (=asking whether causality is compatible with present knowledge of the disease), respectively (Fredricks and Relman, 1996). Such an extension avoids an important category of empiricism, namely the notion of “falsification” and the consequential dump of a theory in favor of another (Buchanan et al., 2006). Revised criteria rather allow the inclusion of other scenarios to explain the absence of viral sequences in some SCCs. For instance, considering the importance of cell–cell communication in a tumor microenvironment, it is also conceivable that a small fraction of HPV-positive cancer cells secrete paracrine acting vesicles (e.g., exosomes) that may stimulate abnormal proliferation of surrounding virus-negative cells and in turn tumor growth (Dalla Pozza et al., 2017; Harden and Munger, 2017). However, the strongest arguments for at least an obligatory initial function of certain viruses in cancer development are vaccination strategies where the prevention of tumor formation is the most stringent read-out criteria for the success of a vaccine (Moore and Chang, 2017).

Hence, the question of an etiological or merely an opportunistic role of beta-HPV types in skin cancer development is accomplished in a concept, referred as the “hit-and-run” mechanism of microbe/virus induced carcinogenesis (reviewed in Galloway and McDougall, 1983; Iwasaka et al., 1992; Niller et al., 2011; Hufbauer and Akgül, 2017). Alternatively, the term “indirect carcinogen” was suggested (Mossman et al., 2004), but this semantically points more toward a substance or an irradiation event as environmental carcinogen than to an infectious agent and confuses the notion of prophylactic vaccination to prevent tumor formation induced by an “indirect” carcinogen. However, a “hit-and run” model of gene regulation is not without precedent. As already suggested several decades ago (Schaffner, 1988), recent experiments indeed showed that certain transcription factors (e.g., bZIP1) epigenetically change the histone code after transient binding (“hit”) that allows the assembly of transcription complexes to continue gene expression after its dissociation (“run”) (reviewed in Varala et al., 2015). Whether certain HPV types also use this mechanism remains to be elucidated.

Nevertheless, numerous seroepidemiological reports support the notion of an association between beta-HPV infection and SCC or its precursors (Andersson et al., 2012; Chahoud et al., 2016), despite the occasional absence of the viral DNA within malignant lesions. Here, another distinct property between mucosal and cutaneous HPV types may account for the occasional loss of viral DNA during skin carcinogenesis, namely the physical state. In contrast to anogenital cancer, cutaneous HPVs do not integrate into the host genome and persist as extrachromosomal elements in a defined copy number. Since HPVs completely depend on the host cell replication machinery, UV-induced DNA damage and the mode of accompanying repair mechanisms may disrupt the maintenance of episomal DNA during viral persistence (reviewed in Doorbar, 2016; Bristol et al., 2017; Wendel and Wallace, 2017).

## How Can a “Hit-and-Run” Mechanism Be Explained?

Accepting tentatively an etiological function of beta-HPV in conjunction with UV exposure in NMSC development, several studies have identified different mechanisms that may reasonably explain a viral contribution to a “hit-and-run” mode of carcinogenesis. Cutaneous HPVs infect and persist as extrachromosomal genomes in basal keratinocytes especially around hair follicles (Weissenborn et al., 2012) which are a reservoir for stem cells with different properties (Jaks et al., 2010). During wound healing (Donati et al., 2017) and skin cancer development in transgenic mice expressing the complete early region of HPV8 (Lanfredini et al., 2017), these cells become activated and start to proliferate.

The main targets of mucosal HPVs are p53 and pRB, which is sufficient to transform the host cell and initiate uncontrolled proliferation (see Doorbar et al., 2012 and references herein), by impairing many downstream pathways such as cell cycle arrest, metabolism, apoptosis, and cross-talks to the immune system (Harris and Levine, 2005; Rozan and El-Deiry, 2007; Moody and Laimins, 2010). In contrast, cutaneous papillomaviruses developed several alternative strategies to interfere with their host cells: instead of binding E6AP to facilitate p53 degradation as alpha type HPVs, E6 of most other HPV genera, amongst them the cutaneous types HPV1 and HPV8, binds to MAML1 to inhibit NOTCH signaling, a feature also shared by animal PVs (e.g., BPV1, MmuPV1, and MnPV) (Brimer et al., 2012, 2017; Meyers et al., 2017). MAML1 together with the histone acetyltransferases p300 and CREBBP can form a transcription complex that activates this pathway (Rozenblatt-Rosen et al., 2012). Amongst its function as a tumor suppressor, NOTCH drives differentiation of keratinocytes and is therefore found to be frequently mutated in cutaneous SCCs (Pickering et al., 2014; South et al., 2014). An impairment of this pathway either by mutations or by intervention of HPV keeps the cell in a proliferative state and promotes tumorigenesis. HPV5 and HPV8 additionally interfere with host cell differentiation by an E6-mediated degradation of p300, which in turn decreases the expression of keratin 1 and 10 and involucrin (Howie et al., 2011).

Furthermore, HPV38 E6 binds p300 thereby preventing p53 acetylation at lysine 382 and blocking p53-mediated apoptosis (Muench et al., 2010). HPV38 E6 can also increase the expression of the N-truncated isoform of p73, lacking a transactivation domain. ΔNp73 in turn competes with p53 activity that may perturb apoptosis and the eradication of damaged HPV-positive cells (Caldeira et al., 2003). A recent study further showed that by targeting p300, HPV8 E6 maintains a proliferating state of the host cell by downregulating the CCAAT/enhancer-binding protein (C/EBPα) and decreasing the expression of microRNA-203, a repressor of ΔNp63 expression (Marthaler et al., 2017). While p63, the master regulator of epithelial stemness, can induce cell cycle arrest and apoptosis like p53 and p73, ΔNp63 can act in a dominant-negative manner and maintain proliferation (reviewed in Candi et al., 2015). Indeed, certain beta-HPVs target central hubs within the cellular network that control differentiation, senescence as well as apoptosis, which reasonably explains the capacity of these viruses to stimulate proliferation of undifferentiated cells. Such effects on tissue homeostasis are of profound importance in NMSC development, particularly when HPV-infected skin is cumulatively exposed to UV.

UV exposure under physiological circumstances induces p53 stabilization and activation that leads to a cell cycle arrest and DNA repair or – at higher dosages – to apoptosis (reviewed in Zuckerman et al., 2009). Perturbed p53 function in turn leads to a gradual accumulation of genetically altered cells, thereby promoting the development of NMSC (Feltkamp et al., 2008). While high-risk mucosal types directly target p53 itself after DNA damage, cutaneous beta-HPVs affect a plethora of DDR proteins downstream of p53 (White et al., 2014; Wendel and Wallace, 2017). An important protein that links the ATM/ATR pathway to p53-induced apoptosis is the conserved Ser/Thr kinase HIPK2 (Matt and Hofmann, 2016). Upon severe UV damage, HIPK2 forms a complex with p53 and the CBP acetyltransferase leading to HIPK2-mediated p53 phosphorylation at serine 46 and CBP-mediated p53 acetylation at lysine 382 and finally a strong p53 activation with induction of pro-apoptotic gene expression (reviewed in Bitomsky and Hofmann, 2009; Matt and Hofmann, 2016). As shown for one of the most prevalent HPV types found in skin (de Koning et al., 2009), HPV23 E6 can inhibit HIPK2-mediated phosphorylation of p53 in response to UV damage (Muschik et al., 2011). Intriguingly, HIPK2 also controls the number of stem and progenitor cells in the skin (Wei et al., 2007), the reservoir for cutaneous HPVs (reviewed in Egawa et al., 2015). Moreover, HIPK2-deficient mice show an enhanced susceptibility to develop SCCs (Wei et al., 2007) consistent with decreased levels HIPK2 expression in KAs and SCCs compared to AK (Kwon et al., 2015). Additionally, beta-HPVs can abrogate UV-induced pyrimidine dimer excision (Giampieri and Storey, 2004) or target the pro-apoptotic protein Bak for proteolytic degradation (Jackson et al., 2000; Underbrink et al., 2008; Holloway et al., 2015). In any case, the interference with the DDR and pro-apoptotic pathways can promote long-lasting effects on genomic instability and favors the accumulation of damaged cells (reviewed in Wendel and Wallace, 2017).

Moreover, UV-activated EGFR signaling which causes keratinocyte hyperproliferation and hyperplasia of the skin (El-Abaseri et al., 2006) is enhanced by HPV8 E6, thereby contributing to SCC formation (Taute et al., 2017). Furthermore, HPV5 and 8 E7 alter beta-catenin and zona occludens-1 anchor proteins which disturb cellular adherence and tight junctions (Heuser et al., 2016a). Their reorganization imbalances tissue homeostasis and lead to epithelial-mesenchymal transition (EMT) (Polette et al., 2007), also suggesting a role in NMSC development (reviewed in Chen et al., 2016). This is in line with an HPV8 E7-mediated upregulation of metalloproteases that remodel the extracellular matrix (Akgül et al., 2006) to facilitate migration and invasion of HPV8-positive cells (Akgül et al., 2005a). Furthermore, HPV8 E7 upregulates fibronectin, another EMT marker (Lee et al., 2006), by inducing a switch from E-cadherin to N-cadherin expression in suprabasal cell layers of organotypic skin cultures, considered to be a primary event in invasion of carcinoma cells (Heuser et al., 2016b).

As soon as HPV-infected altered squamous cells invade deeper layers and undergo EMT, it is likely that HPV replication, known to be highly dependent on cell differentiation, cannot be maintained (reviewed in Doorbar et al., 2012). Consequently, viral presence is no longer necessary for malignant progression, since the tumor becomes heterogeneous in terms of acquiring additional mutations. Despite different modes of action for mucosal and cutaneous HPVs, the outcome is similar: in the case of cervical carcinoma, HPV is integrated into the host cell genome but maintains the transformed phenotype by continuous E6/E7 expression (reviewed in Doorbar et al., 2012). Conversely, cutaneous HPVs interfere with their host cell apparently in the beginning of the multi-step process of carcinogenesis, finally leading to an intracellular environment counteracting episomal DNA replication. This may explain why lower or no viral loads are detected in SCCs as compared to pre-malignant lesions (Weissenborn et al., 2012). In any case, this is a “dead end” for both mucosal and cutaneous HPVs, since the permissive cycle is interrupted and no new virus progenies can be formed.

## The Importance of Preclinical Models for Infectious Agents – Some General Considerations

Models in bioscience are indispensable to reduce the complexity of a disease manifested in patients, but of course they also have their inherent limitations (Anisimov et al., 2005; Mazzocchi, 2008; Dougherty and Shmulevich, 2012). Moreover, in contrast to the empirical supposition of a working hypothesis as “right” or “wrong,” models can also be tentatively categorized as “relevant” or “irrelevant” for the investigation of a particular scientific question or even a multifactorial disease. Since there is a strong social pressure on basic research to bring reproducible laboratory results into the clinic (Begley and Ellis, 2012), there are indeed many recent initiatives to improve this transfer by validating the relevance and impact of *in vitro* and *in vivo* models with respect of their translational applicability (Denayer et al., 2014; Day et al., 2015; Dolgos et al., 2016; Horvath et al., 2016).

Animal experiments for infectious agents like papillomaviruses are also indispensable (Doorbar, 2016; Christensen et al., 2017) and should reflect as much as possible the molecular and histological key features of a disease as found in patients (reviewed in Greek and Menache, 2013). Nevertheless, besides pragmatic reasons in working with easily managing laboratory animals (e.g., rodents) (reviewed in Hu et al., 2017; Uberoi and Lambert, 2017), there are several other important criteria for validation and predictability to guarantee a successful clinical translation. They can be summarized as follows (Kern, 1982; Greek and Menache, 2013; Denayer et al., 2014):

1. The infectious agents should have the same species specificity, tissue tropism and genome organization as their human etiological counterparts.
2. The natural infection mode should be similar. This is of particular importance with respect of the extent and spread of an infection since it may affect both the time course of seroconversion as well as the pathological outcome of the disease.
3. The infectious agent should accomplish the revised Henle-Koch criteria in terms of etiology (Fredricks and Relman, 1996; Falkow, 2004; Mossman et al., 2004). Here, some restrictions have to be considered, especially in the light of novel insights into the organization of individual human virome that can be changed in a time-dependent manner (Virgin, 2014; Gentile and Micozzi, 2016; Vouga and Greub, 2016).
4. The viral inoculum in an experimental infection should not over stimulate the immune system (Kern, 1982).
5. The animal should be immunocompetent and specific antibodies to the respective agents normally absent before exposure. Although especially inbred rodent systems do not necessarily completely mirror human immunology (reviewed in Mestas and Hughes, 2004), they still allow dissecting virus–host interactions in terms of acute and chronic/persistent infections in correlation with the serological response.
6. Depending on immune surveillance in the case of tumor formation, the same lesions should appear, ideally in the same time frame relative to the median life time of the animals (“face validity”).
7. Since cancer is a multi-factorial process, the animal model should also allow the inclusion of additional non-genetic risk factors for a disease (e.g., immunosuppression, UV exposure), leading to an enhanced tumor formation.
8. Having identified a potential infectious agent, subsequent vaccination of animals should prevent the disease. Hence, preclinical models ideally should develop the same clinical manifestations as found in patients, an important read-out criterion for the success of a vaccine to be transferred into the clinic (Moore and Chang, 2017).
9. To be valid as a preclinical model, a scoring system should be applied that includes the careful selection of the animal species, the degree of reflecting a disease, face validity, complexity, and predictability (Denayer et al., 2014).

## How Do the Current Rodent Models for Cutaneous HPVs Fit to These Criteria?

### Transgenic Mice

Transgenic mice are suitable model systems for most research fields and have also contributed enormously to our mechanistic knowledge about mucosal and cutaneous PVs (reviewed in Doorbar et al., 2015; Lambert, 2016; Santos et al., 2017). Current genome editing technologies make them relatively easy to generate and to manipulate (Rocha-Martins et al., 2015). The transgene, regularly containing the open reading frames (ORFs) of E6, E6/E7 or the complete early region of the selected HPV type is typically expressed under control of the keratin 14 promoter which facilitates constitutive oncogenic expression in cycling epithelial cells. The composition of the ORFs and the varying oncogenic potential of cutaneous HPVs significantly influence the outcome of experiments, e.g., the tumor induction. For example, mice harboring the complete early region of HPV8 spontaneously develop papillomatosis, acanthosis and hyperkeratosis, epidermal dysplasia and, to lower frequency, SCC formation (Schaper et al., 2005). Here, overexpression of the early region leads to a clonal expansion of a population of Lrig1^+^ keratinocyte stem cells in the hair-follicles that is accompanied by a switch of p63 to its ΔNp63 isoform, lacking the N-terminal transactivation domain (Lanfredini et al., 2017). This in turn interferes with the function of p63 and p73 to induce cell cycle arrest and apoptosis. It also impairs the cellular differentiation program and drives proliferation (reviewed in Candi et al., 2015). Due to the involvement of Lrig1^+^ stem cells in wound healing (Donati et al., 2017), it is not surprising that development of papillomatosis could be also accelerated after UV exposure, further suggesting a synergistic effect between HPV8 and exogenous factors (Marcuzzi et al., 2009). Furthermore, via targeting of C/EBPβ, HPV8 E7 also suppresses the expression of the CC chemokine ligand 20 (CCL20), which plays a pivotal role in the recruitment of Langerhans cell precursors into the epidermis (Sperling et al., 2012). Hence, considering all these scenarios, the impact of HPV8 on cell differentiation and innate immunity can reasonably explain how a viral infection commits cells to malignant transformation.

The mechanisms by which cutaneous HPVs interfere with their host cell, e.g., by disturbing p53-connected pathways (White et al., 2014) or the DDR to UV, can lead to different outcomes of experiments (Dong et al., 2008; Hufbauer et al., 2011; Viarisio et al., 2011; Holderfield et al., 2014). For instance, as shown recently for HPV49 E6/E7 transgenic mice, these animals are less susceptible to form UV-induced tumors as compared to mice expressing other beta-HPVs, but are prone to chemically induced carcinogenesis of the upper digestive tract (Viarisio et al., 2016). Moreover, in contrast to the HPV8 model where one UV dose was sufficient to induce skin lesions (Marcuzzi et al., 2009), HPV38 E6/E7 transgenic mice require an extended irradiation protocol (Viarisio et al., 2011), mimicking a more natural situation as found in humans after life-long UV exposure (Maglennon et al., 2014). Tumors obtained in the HPV8 model after a single UV dose lack mutations in the *Trp53* and *Notch1* loci, but these were present after cumulative UV exposure in HPV38 mice. Notably, SCCs appeared much earlier in the latter model when UV irradiation and BRAFi treatment were combined. Intriguingly, although no HRAS and KRAS mutations were found in these tumors, the mitogen-activated protein kinase pathway was activated (Viarisio et al., 2017). Monitoring clinical SCC samples obtained from melanoma patients, it seems that HRAS mutations and the presence of HPV is mutually exclusive (Holderfield et al., 2014), a constellation that is reminiscent of transgenic mice expressing HPV38 E6/E7 constitutively.

Considering the concept of a “hit-and-run” mechanism during tumorigenesis, transgenic models are obviously not fully suitable when used in a conventional way by constitutive transgene expression. However, Viarisio et al. (2018) recently were able to overcome this problem by knocking out the HPV38 E6/E7 cassette via Cre-lox recombination as soon as UV-induced lesions appeared. After this, SCCs continued growing even without oncogene expression, clearly demonstrating that viral presence was no longer required to maintain the tumorigenic phenotype. Moreover, these SCCs acquired a large panel of potential “driver” mutations within *Trp53, Notch1*, and other genes (Viarisio et al., 2018) that can also be observed in human and murine SCCs (Pickering et al., 2014; Chitsazzadeh et al., 2016).

Although transgenic and knock-out models definitely have their justification when investigating potential principles of an *in vivo* virus–host interaction, concerns may be raised as they only reflect parts of the whole picture and do only partially, if at all, fulfill the above mentioned criteria to guarantee a successful clinical translation. The described examples are mostly typical “gain-of-function” studies, where the viral transgene is integrated and controlled by a heterologous promoter, thereby not mimicking the physiological gene dosage and the physical state of the viral DNA which normally persists extrachromosomally in the host cell. Additionally, the encoded transgenic proteins, provided there is no conditional gene expression, are self-tolerant and do not mirror a proper immune response against the corresponding genuine antigens expressed during a natural infection (Walrath et al., 2010). Moreover, according to quantitative trait locus (QTL) mapping analysis for cancer susceptibility loci, inbred strains of mice tremendously vary in their ability to develop tumors (Fleming et al., 2013). Hence, although being extremely helpful, experiments with transgenic mice have to be complemented with natural infection models to get also insight in immunological aspects, particularly allowing the production of appropriate vaccines to prevent NMSC (Vinzón and Rösl, 2015).

### Natural Model Systems

Restricting this overview only to rodent systems, and also considering the above mentioned criteria for a relevant preclinical model, the MmuPV1/ mouse model (reviewed in Hu et al., 2017) is one of the most attractive, since the mouse is not only the best-characterized laboratory animal, but can also be infected by a virus isolated from the same species. Although just discovered in 2011, Xue et al. (2017) recently published a full transcription map of MmuPV1, providing a basis for a further characterization of viral proteins and their function in skin tumor formation. Moreover, first vaccination studies have been carried out in this system (Jiang et al., 2017). However, even with the MmuPV1/mouse model, the setup of a natural infection followed by a complete viral life cycle in a normal and immunocompetent host is unfortunately still not completely fulfilled. In this model, for instance, lesions appear only when infecting mice with high and, in comparison to humans, non-physiological amounts of virus particles at certain sites like the tail and muzzle, while the back skin is relatively resistant (Handisurya et al., 2013, 2014; Wang et al., 2015). This may be attributed to differential expression of MHC molecules, the presence of Langerhans cells and variations in the keratin network (Sundberg et al., 2014; Quigley et al., 2016), supporting the importance of the local immune surveillance (Da Silva et al., 2014) at the region of infection. These differences may also cause inappropriate conditions for virus particle formation that have to be overcome by a high infection rate, since fewer progenies could be detected in back skin than in mucosal tissue, despite equal viral DNA loads in both regions (Cladel et al., 2017). Cutaneous MmuPV1-induced tail papillomas contained high amounts of viral DNA (Uberoi et al., 2016), but no SCCs lacking MmuPV1 DNA are formed in immunocompetent animals that allow to study a “hit-and-run” mechanism as proposed.

**FIGURE 1:**
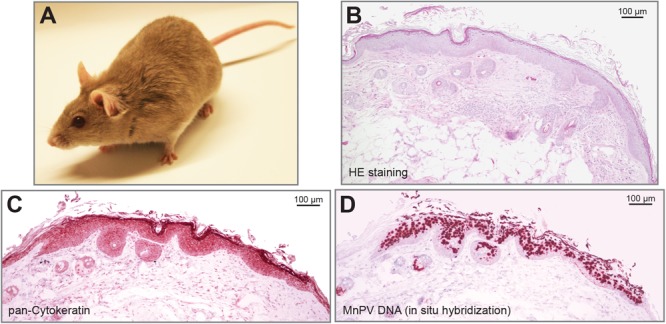
The preclinical animal model *Mastomys coucha* develops MnPV-induced skin lesions. **(A)** A young *Mastomys coucha*. **(B)** HE staining of a dysplastic epithelium at the edge of a spontaneously emerging skin lesion. **(C)** Pan-cytokeratin staining shows the dysplasia of the epithelium. **(D)** *In situ* hybridization reveals squamous cells positive for MnPV DNA.

Another preclinical model that is used to explore the impact of cutaneous papillomaviruses on skin carcinogenesis is the African multimammate mouse *Mastomys coucha*, formerly taxonomically classified as *Mastomys natalensis* (**Figure 1**; Kruppa et al., 1990). These animals belong to the Muridae family and are naturally infected with MnPV and McPV2, which – like cutaneous and genital HPVs – infect epidermal and mucosal tissues, respectively (Muller and Gissmann, 1978; Nafz et al., 2008). MnPV can spontaneously induce epithelial lesions of the skin (mainly papillomas and KAs), while McPV2 causes anogenital lesions like condylomata at the anus, vulva, and penis, respectively. Similar to cutaneous HPVs, MnPV and McPV2 persist as episomes without any indication of integration but – depending on the type of lesion – in different copy numbers (Nafz et al., 2008). Considering the natural infection mode, *M. coucha* acquire the virus early after birth, as MnPV DNA is found in the skin of four-week-old animals where at the same time seroconversion is taking place (Schäfer et al., 2011).

Similar to cutaneous HPVs, the MnPV genome lacks an ORF for E5 (de Villiers et al., 2004) while parts of the E6, E1, and L1 genes are phylogenetically related to HPV types associated with EV (Tan et al., 1994). MnPV-induced benign lesions do not spontaneously regress but have the capacity to transform to SCCs (Reinacher et al., 1978; Vinzón et al., 2014). The exo-/endophytic keratinized lesions contain high numbers of episomal virus genomes (**Figures 1B–D**) and – similar to cutaneous HPVs – do not show any sign for integration (Amtmann et al., 1984; Hasche et al., 2017). As the animals are immunocompetent, E2-specific antibodies serve as an early infection marker found already in four-week-old animals, while L1-specific antibodies appear later, correlating with the appearance of skin lesions (Schäfer et al., 2011). Having a virus-free colony of *M. coucha* as control, the animals also served as preclinical model for the development of MnPV-specific vaccination strategies both under normal and immunosuppressed conditions (Vinzón et al., 2014). According to the relevance criteria for animal systems summarized above, *M. coucha* permits the follow-up of the complete infection cycle, starting from primary infection until the development of lesions that are not restricted to a certain body area (Nafz et al., 2007). Transcriptome analysis of skin tumors lead to the identification of various mRNA isoforms of MnPV (Salvermoser et al., 2016). Moreover, *M. coucha*-derived cell lines (Hasche et al., 2016) can be used to test the effects of additional factors on viral transcription and replication, making previous drawbacks compared to mouse systems more and more negligible.

**FIGURE 2:**
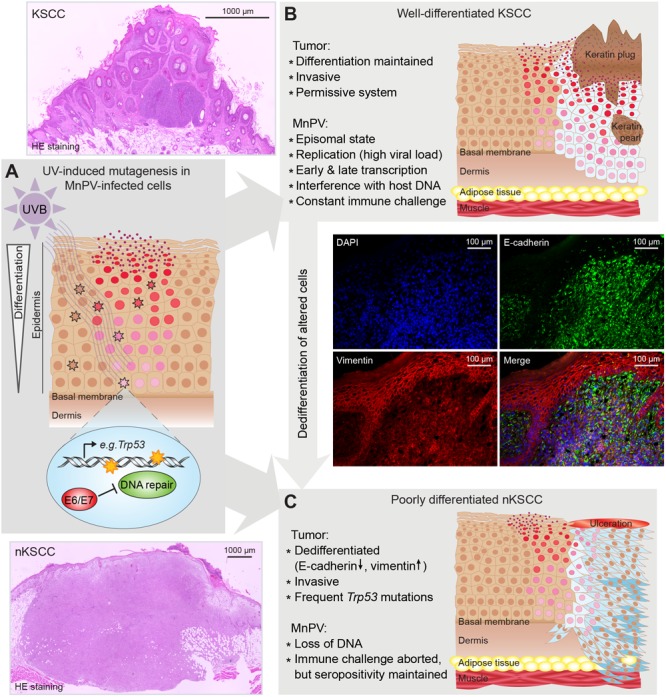
Suggested “hit-and-run” model based on experiments with *Mastomys coucha*. **(A)** Virus replication and virion formation depend on differentiating squamous cells and are favored by UV-induced hyperproliferation. UV induces photoproducts, e.g., in *Trp53*. In uninfected cells, damages are repaired. In infected cells, MnPV reduces chromosomal stability and inhibits DNA repair leading to an accumulation of mutations. **(B)** Altered squamous cells become neoplastic (light blue) and start forming a well-differentiated keratinizing SCC, still representing a permissive system that allows viral replication and formation of virions. **(C)** When neoplastic squamous cells accumulate further mutations (dark blue), a dedifferentiated phenotype (characterized by a switch from E-cadherin to vimentin expression) is acquired, forming a poorly differentiated, non-keratinizing SCC (nKSCC). MnPV cannot replicate in this intracellular environment, resulting in a loss of viral DNA. (Scheme modified from Hasche et al., 2017).

Notably, the *M. coucha* model resembles in many characteristics UV-induced NMSC development in humans. In a large long-term study, the animals were irradiated with UV doses that are comparable to different areas of the world (Hasche et al., 2017). Naturally infected animals developed SCCs significantly more often than virus-free controls. Some of these SCCs were well differentiated and keratinizing (KSCCs), containing high amounts of extrachromosomal and transcriptionally active MnPV genomes as observed previously in spontaneous MnPV-induced tumors (Amtmann et al., 1984; Vinzón et al., 2014), whereas poorly differentiated and non-keratinizing SCCs (nKSCCs) only contained low amounts or even lacked viral DNA. Histologically KSCCs were similar to human AKs and Bowen’s disease (Majores and Bierhoff, 2015) which also contain high viral loads whereas SCCs usually lack HPV DNA (Weissenborn et al., 2005). However, irrespective of the tumor type, all tumor-bearing animals developed antibody responses against the viral L1 capsid protein, providing evidence for preceding infections, which again is seen in SCC patients (Andersson et al., 2008). Notably, although showing the same time course of induction, the histology of UV-induced SCCs was profoundly influenced by the dose, with KSCCs mostly developing in the lowest dose group and nKSCCs preferentially under higher doses, pointing to the cumulative effect of UV causing human SCCs (Leiter and Garbe, 2008). Consequently, nKSCCs more often harbored p53 mutants, especially at two hot-spots also known from human SCCs (Pickering et al., 2014) and incapable of activating expression of downstream targets. As shown in mice before, a loss of functional p53 leads to less differentiated tumors (Flores et al., 2016; Page et al., 2016) and induces cell migration and degradation of the extracellular matrix (reviewed in Muller et al., 2011). Since MnPV replication depends – like cutaneous HPVs – on cell differentiation (reviewed in Doorbar et al., 2012), this constellation can explain the loss of viral genomes in these tumors by acquiring additional “driver” mutations (e.g., *Trp53*) (**Figure 2**). Moreover, taking into account that more DNA damages have been detected in MnPV-positive skin compared to MnPV-negative skin, this model provided for the first time evidence for a “hit-and-run mechanism” in a natural infection system (Hasche et al., 2017).

Notably, a recent *in vitro* study showed that MnPV E6 does not interact with E6AP, a prerequisite for p53 degradation, but – like other cutaneous human and animal PVs – interacts with MAML1 to inhibit NOTCH signaling (Brimer et al., 2017). The same was reported for MmuPV1 E6, which also inhibits differentiation of keratinocytes to keep the host cell in a proliferative state (Meyers et al., 2017). However, to examine the effect of UV exposure in the context of a cutaneous PV infection requires more systematic studies, particularly to understand how a single UV dose, shown to have immunosuppressive effects (reviewed in Norval and Halliday, 2011), makes wildtype mice susceptible to MmuPV1-induced tumors that can progress to SCCs (Uberoi et al., 2016), while in other systems more frequent exposure is necessary. This is an important point, especially for immunosuppressed OTRs and the high incidence of NMSC in these patients. However, the fundamental question is still how cutaneous PVs contribute to tumor initiation and what kind of selection mechanisms account for SCC formation and the loss of viral DNA.

## Current Work and Perspectives on Vaccination Against Cutaneous HPVs

The strongest argument for an etiological role of a virus in cancer development is the application of a prophylactic vaccine that prevents tumor formation. In the case of anogenital tumors, three HPV vaccines (Cervarix, Gardasil, and Gardasil 9), consisting of L1 (the major papillomavirus virion protein)-based virus-like particles (VLPs) are currently licensed (Pogoda et al., 2016). They are targeted against two, four, or nine mucosal HPV types (HPV6, 11, 16, 18, 31, 33, 45, 52, and 58), respectively, and – regardless of being very effective – they present limitations, making the development of broad protective second-generation HPV vaccines necessary. Most of the mechanistic studies showed that vaccine-induced neutralizing antibodies are the primary mediators of the elicited response, protecting from a subsequent infection by the targeted HPV types and therefore conferring immunity (Schiller and Lowy, 2012). Since the response has been shown to be largely type-specific and cross-reactivity among different HPV types is almost absent, these vaccines target, at best, the high risk types that cause 90% of the cervical cancer and no cutaneous types. Indeed, there are more than 40 HPV types plausible of being targeted by a vaccine to prevent human diseases: mucosal high-risk HPVs implied in the pathogenesis of cervical and other cancers (HPV16, 18, 31, 33, 35, 39, 45, 51, 52, 56, 58, 59, 68), additional mucosal types which cause genital warts (HPV6 and 11), several beta HPV types potentially linked to NMSC (HPV5, 8, 9, 12, 14, 15, 17, 19, 20, 21, 22, 23, 24, 25, 36, 37, 38, 47, 49) and the different alpha (HPV2, 3, 10, 27, 28, 57), gamma (HPV4, 60, 65), mu (HPV1, 63), and nu (HPV41) types that induce different lesions, being a special burden in children and immunocompromised patients. Given the potential benefits of covering so many different HPV types, a broad-protecting prophylactic HPV vaccine is a rational goal for second-generation vaccine development.

In this context, the papillomavirus minor capsid protein L2 contains a region with a major cross-neutralizing epitope that has been the main immunogen for second-generation vaccines (Gambhira et al., 2007). The goal of developing an L2-based vaccine is to generate a single- or oligo-valent antigen with a distinctively larger spectrum of protection against genital and cutaneous HPVs than the current formulations (Pouyanfard and Muller, 2017). Given that L2-derived linear peptides induce antibody levels which are several orders of magnitude lower that those induced by L1 VLP vaccines, different strategies had been explored in order to achieve a successful immune response. Among these, good responses were found by formation of L2 concatemers (Jagu et al., 2009), conjugation to a T-helper epitope and a TLR ligand (Alphs et al., 2008), display on structures like bacteriophages (Tumban et al., 2011) or on adeno-associated viruses (Nieto et al., 2012), chimeric HPV L2 peptide/L1-VLPs (Schellenbacher et al., 2009), integration into the thioredoxin active site (Rubio et al., 2009) or lipidation and fusion to FcγR-targeting scaffold (Zhang et al., 2016). These approaches have shown elicitation of neutralizing antibodies with a broad range of cross-neutralization, setting the proof-of-principle for the application of this approach.

Several vaccine formulations, either L1- or L2-based, have shown the development of antibodies neutralizing to PV cutaneous types (summarized in **Table 1**), either by targeting them directly or as a cross-protection. In particular, two studies have shown the potential of these vaccines to effectively prevent skin lesions in preclinical models. When *M. coucha* was used as a model, a VLP-based vaccine against a cutaneous papillomavirus could prevent spontaneous skin tumor formation both under normal and immunosuppressed conditions (Vinzón et al., 2014). As mentioned, MnPV-infected *M. coucha* resemble many characteristics of cutaneous HPVs and the human situation, like the natural and persistent infection early in life (Schäfer et al., 2011). Assessing the efficacy of a VLP-based vaccine on either previously or newly established infections, a long-lasting response could be shown, characterized by the induction of neutralizing antibodies that confer protection against both benign and malignant skin tumors even in immunosuppressed animals (Vinzón et al., 2014). Another novel finding of this study was that protection involves the maintenance of a low viral load in the skin by an antibody-dependent prevention of virus spread and that it is effective in preventing tumor formation even in individuals already infected at the time of vaccination. These results offer evidence that VLPs elicit an effective immune response in the skin even under immunosuppressed conditions, irrespective of the infection status at the time of vaccination, which sets the basis for a future clinical implementation of a vaccine against cutaneous HPV-induced tumors to prevent SCCs, especially in OTRs.

**Table 1:** Preclinical studies showing reactivity of HPV vaccines to cutaneous types/challenge.

Immunogen scaffold/type	PV immunogen	Cutaneous HPV types neutralized by the vaccine *in vitro*	HPV types neutralized by cutaneous challenge	Projected vaccine development	Reference
VLPs	L1 from HPV 5, 8, and 92	HPV 5	ND	Unknown	Handisurya et al., 2009

Capsomers/VLPs	L1 from HPV 2, 27, and 57	HPV 2, 27, and 57	ND	Unknown	Senger et al., 2010

VLPs	L1 from MnPV	MnPV	MnPV	No	Vinzón et al., 2014

Lipopeptide (P25-P2C-HPV)	HPV16 L2(17–36)	HPV 5, BPV 1	HPV 16 and 45	Unknown	Alphs et al., 2008

L2 concatemers	Fusion of the L2(11-88) region of HPV types 6, 16, 18, 31, 39, 51, 56, and 73	HPV 3, 5, 8, 23, 27, 38, 57, 76	ND	Unknown	Kwak et al., 2014

Lipidated L2-repeat fusioned to an anti-hFcγRI scFv	HPV16 L2(17–36)	HPV 2 and 5	ND	Unknown	Zhang et al., 2016

Chimeric VLPs	HPV16 L2(17–36)	HPV 2, 3, 5, 27, 76	ND	Being produced under cGMP for clinical trials by the NCI PREVENT program (Shi et al., 2001).	Schellenbacher et al., 2009, 2013

Chimeric VLPs	HPV17 L2(17–36)	HPV 5, 20, 24, 38 and 96	HPV 5	Unknown	Huber et al., 2017

Chimeric VLPs	HPV 58 L2(16–37)	HPV 2, 5, 27 and 57	ND	Unknown	Chen et al., 2017

Fusion to bacterial flagellin	L2(11–200), L2(11–88) and/or L2(17–38) of HPV 6, 16, 18, 31, 39 and 52	ND	HPV 6, 16, 18, 31, 39, 52 and CRPV	Unknown	Kalnin et al., 2014, 2017

*Pyrococcus furiosus* thioredoxin with L2 octamer	L2(20–38) from HPV 6, 16, 18, 31, 33, 35, 51, and 59	HPV 3, 4, 5, 10, 38, 63, 76, 92, 95, and 96	ND	Close to cGMP production for a planned human trial (Pouyanfard and Muller, 2017).	Pouyanfard et al., 2017; Spagnoli et al., 2017

PP7/MS2 bacteriophage VLPs	HPV16 L2(20–29), (17–31), (14–40) and (14–65)	ND	HPV5	Being developed by the company Agilvax with DMID/NIAID/NIH support (Pouyanfard and Muller, 2017).	Tumban et al., 2012

Naked-DNA, fusion to calreticulin	MmuPV1 E6 and E7 and L2(11–200)	ND	ND	Being produced under cGMP for clinical trials by the NCI PREVENT program (Shi et al., 2001).	Jiang et al., 2017

DNA vaccines were also explored in the context of cutaneous HPV infection. A recent study (Marcuzzi et al., 2014) showed that a vaccine consisting of HPV8 E6 DNA can generate a specific cellular response in roughly half of the vaccinated animals and partially prevent papilloma formation in those which developed HPV8-E6-specific T cell immunity as demonstrated by ELISPOT. Regarding L2 vaccines, a simple HPV16-L2 DNA vaccine showed no induction of neutralizing antibodies and therefore no cross-reactivity potential (Hitzeroth et al., 2009). In contrast, another vaccine composed of HPV16-L2/E6/E7 fused to human calreticulin (hCRT) as a strategy to enhance MHC class I presentation, induced both E6/E7-specific T-cell responses and L2-specific neutralizing antibodies, showing both therapeutic and prophylactic potential (Kim et al., 2008). hCRT-E6E7L2 conferred partial protection in an *in vivo* neutralization assay (Jiang et al., 2017), but its cross-neutralization activity has not been yet reported. Furthermore, when exploring the vaccine in an immunosuppressed setting, hCRT-E6E7L2 vaccination maintained cellular immune reactivity in CD4^+^ T cell-depleted mice but lost the ability to induce a humoral response (Peng et al., 2014). hCRT-E6E7L2 is currently produced under cGMP for clinical trials by the NCI PREVENT program (Schellenbacher et al., 2017).

The recent development in the MmuPV1 model allowed vaccination studies also in this animal system (Jiang et al., 2017). An hCRT-MmuPV1E6E7L2 DNA vaccine induced strong mE6 and mE7 CD8^+^ T cell responses and anti-L2 antibodies. In addition to the vaccine-induced antibody titers, also a robust anti-L1 response could be detected regardless of the papilloma status of the animals, probably due to the inoculum used for the experimental infection. Remarkably, persistent papillomas disappeared within 2 months after treatment and the virus could no longer be detected. Given the design of the vaccine, the importance of neutralizing anti-L2 antibodies cannot be dissected, as the anti-L2 response is concomitant to the E6 and E7 responses.

Therefore, the effectivity of pure L2 vaccines in preventing skin tumors is yet to be studied in a complete preclinical model. One important issue to be answered is whether L2 vaccination can elicit enough neutralizing antibodies to confer protection against tumor formation, especially against non-cognate HPV types, since L2 responses are known to be lower than their L1 counterparts. In that context, even when sera display titers below detection limit by *in vitro* neutralization assays and *in vivo* neutralization assays after passive transfer, vaccinated animals still show complete protection against infection, which points to the fact that very low titers are indeed sufficient to confer immunity (Kalnin et al., 2017).

## Conclusion

Current data strongly support an involvement of cutaneous HPVs in the pathogenesis of NMSC. It is clear that a vaccine targeting a broader spectrum, including mucosal and cutaneous HPVs would be beneficial for certain patients, especially those who are immunosuppressed. Since cutaneous HPV infections are acquired very early in life, vaccination in infancy seems to be most appropriate. The other option would be the vaccination of immunocompromised individuals, for example patients awaiting organ transplantation. By eliminating one of the main risk factors (HPV infection), the incidence of NMSC should be reduced. Clinical development of vaccines that could achieve this goal is currently being pursued.

## Author Contributions

All authors listed, have made substantial, direct and intellectual contribution to the work, and approved it for publication.

## Conflict of Interest Statement

The authors declare that the research was conducted in the absence of any commercial or financial relationships that could be construed as a potential conflict of interest.

## Footnotes

1 https://pave.niaid.nih.gov

## Abbreviations

AK: actinic keratosis
ALK: ALK Receptor Tyrosine Kinase
ATM: ataxia telangiectasia mutated
ATR: ataxia telangiectasia and Rad3-related protein
BPV1: bovine papillomavirus 1
BRAFi: Inhibitors of the B-Raf Proto-Oncogene
CDKN2A: cyclin-dependent kinase inhibitor 2A
cGMP: current good manufacturing practice
CKIT: KIT proto-oncogene receptor tyrosine kinase
CREBBP: CREB binding protein (CREB, CAMP responsive element binding protein 1)
DDR: DNA damage response
E6AP: Ubiquitin Protein Ligase E3A
EGFR: epidermal growth factor receptor
FAT1: FAT atypical cadherin 1
FGFR3: fibroblast growth factor receptor 3
HIPK2: homeodomain-interacting protein kinase 2
HRAS: Harvey rat sarcoma virus oncogene
KA: Keratoacanthoma
KRAS: Kirsten rat sarcoma viral oncogene homolog
MAML1: mastermind like transcriptional coactivator 1
McPV2: *Mastomys coucha* papillomavirus 2
MHC: major histocompatibility complex
MmuPV1: Mus musculus papillomavirus type 1
MnPV: *Mastomys natalensis* papillomavirus
NMSC: non-melanoma skin cancer
OTRs: organ transplant recipients
PIK3CA: Phosphatidylinositol-4,5-Bisphosphate 3-Kinase Catalytic Subunit Alpha
pRb: RB transcriptional corepressor 1
Pten: phosphatase and tensin homolog
SCC: squamous cell carcinoma
TLR: Toll-like receptor
TP53: tumor protein P53 (human)
*Trp53*: transformation related protein 53 (mouse)
